# Biallelic expansion in *RFC1* as a rare cause of Parkinson’s disease

**DOI:** 10.1038/s41531-021-00275-7

**Published:** 2022-01-10

**Authors:** Laura Kytövuori, Jussi Sipilä, Hiroshi Doi, Anri Hurme-Niiranen, Ari Siitonen, Eriko Koshimizu, Satoko Miyatake, Naomichi Matsumoto, Fumiaki Tanaka, Kari Majamaa

**Affiliations:** 1 Research Unit of Clinical Neuroscience, Medical Research Center Oulu, Oulu University Hospital and University of Oulu, Oulu, Finland; 2 Department of Neurology, Oulu University Hospital, Oulu, Finland; 3 Clinical Neurosciences, University of Turku, Turku, Finland; 4 Department of Neurology, Siun Sote North Karelia Central Hospital, Joensuu, Finland; 5 Department of Neurology and Stroke Medicine, Yokohama City University Graduate School of Medicine, Yokohama, Japan; 6 Department of Human Genetics, Yokohama City University Graduate School of Medicine, Yokohama, Japan; 7 Clinical Genetics Department, Yokohama City University Hospital, Yokohama, Japan

**Keywords:** Parkinson's disease, Translational research, Medical genetics

## Abstract

An intronic expansion (AAGGG)_exp_ in the *RFC1* gene has recently been shown to cause recessively inherited cerebellar ataxia, neuropathy, and vestibular areflexia syndrome and, furthermore, a few patients with ataxia and parkinsonism have been reported. We investigated 569 Finnish patients with medicated parkinsonism for *RFC1* and found biallelic (AAGGG)_exp_ in three non-consanguineous patients with clinically confirmed Parkinson’s disease without ataxia suggesting that *RFC1*-related disorders include Parkinson’s disease as well.

The replication factor C complex is a five-subunit ATPase required for DNA replication and repair. The gene encoding subunit 1 (*RFC1*) has been identified as a frequent cause of cerebellar ataxia, neuropathy and vestibular areflexia syndrome (CANVAS) and late-onset ataxia^1,2^. The causative mutation is a biallelic pentanucleotide repeat expansion (AAGGG)_exp_ in the intronic region of *RFC1*, but its functional consequences are not yet known. The expansion explains 90 % of CANVAS and up to 14 % of adult-onset ataxias^3^. The (AAGGG)_exp_ is associated with a core haplotype^1,2,4^ and it has been estimated that the age of the ancestral haplotype is >25,000 years^2^.

Since the identification of (AAGGG)_exp_, other phenotypes, such as cerebellar and parkinsonian type multiple system atrophy (MSA)^5–7^, have been reported in association with *RFC1*. Involvement of dopaminergic neurons in the striatum has been described in a patient with CANVAS and features of Lewy body dementia^4^. Furthermore, one patient with cerebellar ataxia, vestibulopathy and levodopa responsive lower body parkinsonism and another patient with the clinical triad of CANVAS and levodopa responsive parkinsonism have been reported^7,8^. Here we report on screening of a population-based cohort of Finnish patients with medicated parkinsonism for *RFC1* (AAGGG)_exp_.

We found nine subjects with the homozygous (AAGGG)_exp_-associated core haplotype among 569 patients with medicated parkinsonism (Supplementary Table 1) and two out of 269 controls. Neither of the controls, but three of the 9 patients, harbored biallelic (AAGGG)_exp_ in *RFC1* (Fig. 1). The number of repeated units varied from 144 to 820 (Supplementary Table 2, Supplementary Fig. 2). The three patients fulfilled the criteria of Parkinson´s disease (PD)^9^, none had ataxia and one did not have any of the characteristic features of CANVAS. Analysis of exome sequencing data excluded contributing mutations and copy number variants in known PD genes. Biallelic (AAGGG)_exp_ was not found in the remaining 560 patients, who did not have the homozygous core haplotype.

**Fig. 1:**
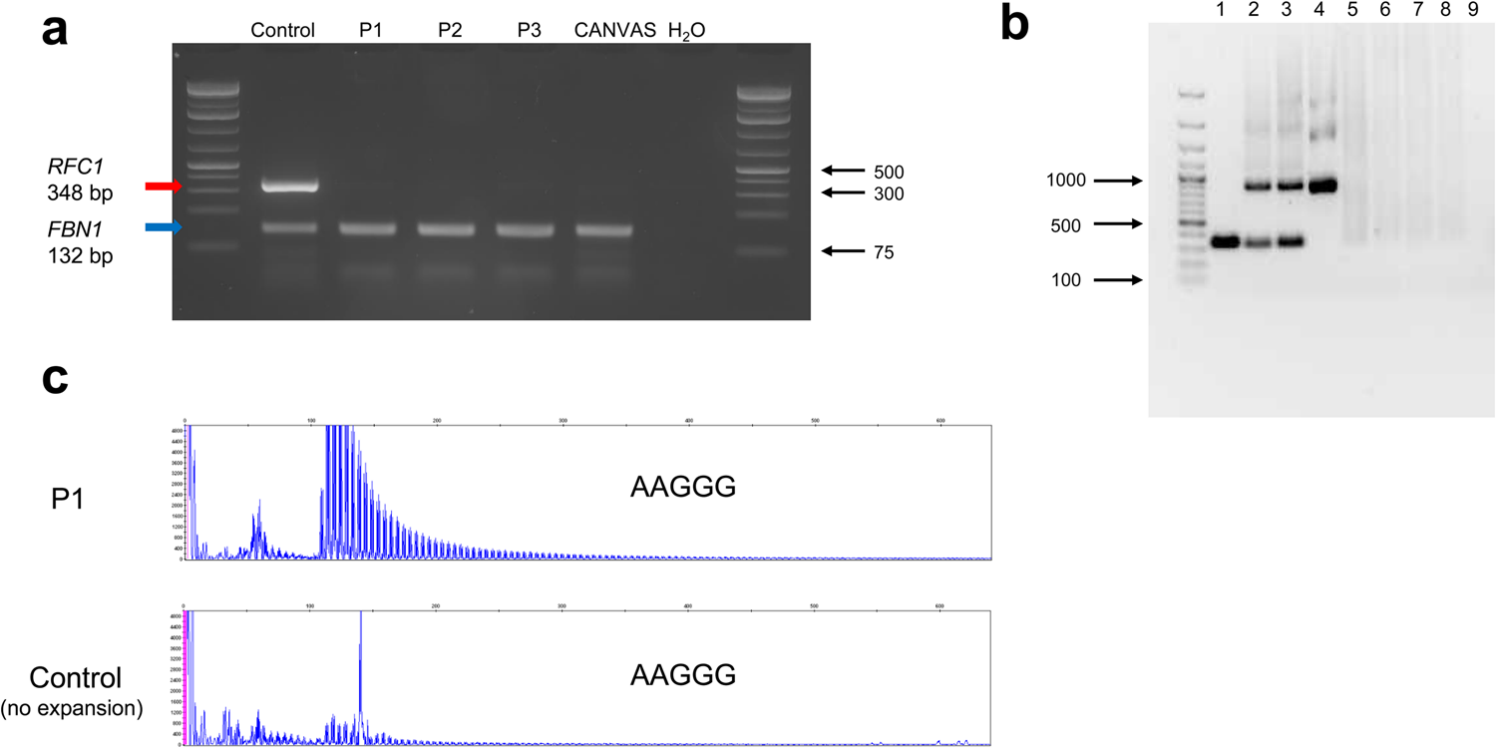
Detection of (AAGGG)exp in RFC1. **a** Multiplex PCR of *RFC1* and *FBN1* shows no *RFC1* PCR product in the region of interest in the three patients with PD (P1-P3) or CANVAS. The gel derives from the same experiment. **b** XL-PCR amplification of *RFC1* carried out with *Phire II* Hot Start DNA polymerase. Lanes 1–4, healthy controls with normal fragment size variation; lanes 5–7, patients 1–3; lane 8, patient with CANVAS and biallelic (AAGGG)_exp_; lane 9, H_2_O. The gel derives from the same experiment. **c** Electropherogram resulting from repeat-primed PCR of patient 1 harboring the biallelic (AAGGG)_exp_ and a control without the expansion.

An experienced neurologist (J.S.) examined patients 1 and 2 at 8 a.m. in both off and on phases, while patient 3 was not available for the clinical study but J.S. had examined her during the preceding year. The extrapyramidal signs were asymmetrical and responsive to levodopa in the three patients and beta-CIT-SPECT imaging at the time of PD diagnosis had revealed markedly asymmetrical dopamine transporter depletion in the putamina (Supplementary Fig. 1). There was some phenotypical variation (Table 1), but the clinical impression was that neither the presentation nor disease course differed from those in other PD patients.

**Table 1:** Clinical characteristics of the three patients with Parkinson’s disease and with biallelic (AAGGG)_exp_ in *RFC1*.

Patient	P1	P2	P3
Sex	Male	Male	Female
Age (years)	73	69	64
Age at onset (years)	65	59	51
Family history	Negative	Negative	Negative
Phenotype	Akinetic-rigid	Tremor-dominant	Tremor-dominant
Dyskinesias	No	No	Yes
Dystonia	No	No	Yes
Hallucinations	Yes	Yes	No
RBD	Yes	Yes	NA
Tendon reflexes	Absent	Brisk knee jerks	Normal
Plantar responses	NA	Negative	Negative
Orthostatic test	Positive	Positive	Negative
MMSE	20/30	25/30	NA
UPDRS I	5	8	NA
UPDRS II	13	16	NA
UPDRS III off	31	31	37
UPDRS III on	16	22	15
UMSARS I	12	13	NA
UMSARS II	14	17	NA
Modified Hoehn & Yahr	3	2.5	3
Chronic cough	No	Yes	Yes
Polyneuropathy	No	Sensory, axonal	Sensory and motor, axonal
HIT	Negative	Positive	NA
Oscillopsia	No	Subjective	NA
Eye movements	Saccadic hypermetria	Normal	Normal
SARA	8	7.5	NA

HIT, Head Impulse Test; MMSE, Mini Mental State Examination; NA, Not Available, RBD, REM Sleep Behavior Disorder; SARA, Scale for the Assessment and Rating of Ataxia; UMSARS, Unified Multiple System Atrophy Rating Scale; UPDRS, Unified Parkinson’s Disease Rating Scale.

Patient 1 (Supplementary video 1) is a 73-year-old man with onset of extrapyramidal symptoms at age 65 years. Computed tomography showed minimal frontotemporal cortical atrophy. The symptoms were quite well controlled for the first four years, after which the symptoms have deteriorated slowly. Clinical examination revealed a mild cognitive impairment with a marked ideomotor apraxia. He was independent in all activities of daily living, but his driving licence has been revoked because of cognitive difficulties. Ropinirole has been discontinued because of hallucinations. Autonomic dysfunction was deemed to be mild to moderate except for marked orthostatism. His comorbidities were arthrosis and lumbar spinal stenosis.

Patient 2 (Supplementary video 2) is a 69-year-old man with onset of extrapyramidal symptoms at age 59 years. The symptoms were easily controlled for the first nine years, but during the last year they have become more severe. Mild cognitive impairment was observed, and computed tomography showed minimal frontotemporal cortical atrophy. Pramipexole has been discontinued because of hallucinations. Patient 2 reported a short vertigo upon turning his head and vestibular areflexia was verified in a neuro-otologic examination. Autonomic dysfunction was deemed to be mild to moderate except for marked orthostatism. He has had a coronary bypass operation, an implanted cardiac pacemaker, and type 2 diabetes.

The blood pressure of patients 1 and 2 fell below 80/50 mmHg (sitting) after the administration of 250 mg of soluble levodopa. Patient 2 experienced a clinical worsening, even as his extrapyramidal signs clearly abated and a similar, although milder, effect was observed in patient 1.

Patient 3 is a 64-year-old woman with onset of extrapyramidal symptoms at age 51 years. The symptoms of PD have steadily progressed. She has been deemed indicated for deep brain stimulation, but the procedure has been deferred. Brain magnetic resonance imaging has been normal (Supplementary Fig. 1) and neuropsychological evaluation has not revealed cognitive impairment. Patient 3 has tolerated only a relatively low dose of pramipexole. She has experienced frequent falls and her autonomic dysfunction has been considered mild. Her comorbidities included hypertension and coronary heart disease.

The phenotype associated with the expansion in *RFC1* is multisystemic including cerebellar, neuropathic, autonomic, extrapyramidal, cognitive and even pyramidal signs^3–8^. Biallelic (AAGGG)_exp_ in *RFC1* commonly manifests as CANVAS or late-onset ataxia with chronic cough^3^. We found that none of our patients had ataxia and that one patient (P1) was lacking all the three core features of CANVAS. The expansion in *RFC1* has also been found in occasional patients with MSA, in a patient with CANVAS and levodopa responsive parkinsonism and in a patient with features of Lewy body dementia^4–8^. Bradykinesia has been reported in 26 % of ataxic *RFC1* patients and it co-occurs with autonomic dysfunction yielding MSA-C phenotype in 19 % of the patients^7^. Only one of the previously reported MSA cases has had an unambiguous levodopa response and most of them appear to have had a more severe phenotype and a more aggressive disease course compared to our patients^5–7^. Autonomic dysfunction was manifested in two of our patients as moderate to severe orthostatic hypotension that was not present in the third patient. Autonomic dysfunction was not a central feature in any of our patients making the phenotype inconsistent with MSA.

We found that the clinical phenotype of the three patients was consistent with PD and that their extrapyramidal symptoms unambiguously responded to levodopa. Patient 3 resembled a previous case of parkinsonism with biallelic *RFC1* (AAGGG)_exp_ in levodopa response, age of onset, dopamine transporter imaging and the presence of chronic cough^8^. However, the disease course appears to have been considerably milder in the previous patient, as her neuropathy was restricted to sensory fibers and, most importantly, she had ataxia^8^, which was not present in our patients. Another patient has been reported with levodopa-responsive lower body parkinsonism and with incomplete CANVAS^7^. Comparison of these five cases suggests that extrapyramidal features are variable in patients with *RFC1* expansion and that some patients fulfill the clinical criteria of PD.

The three patients were from the province of North Karelia. The national registry that was used to identify patients included 797 subjects with medicated parkinsonism and with a residence in North Karelia. Samples were received from 161 subjects and three patients were found with biallelic *RFC1* (AAGGG)_exp_ giving a frequency of 1.9 % (0.4–5.4 %; 95 % confidence interval). Intriguingly, the prevalence of PD is higher in North Karelia than elsewhere in Finland^10^, which may at least partly be attributed to factors related to geographically clustered genetic structure of the Finnish population^11^. Biallelic (AAGGG)_exp_ in *RFC1* may thus be one of the most common genetic causes of PD in Finland, at least in North Karelia.

The frequency of the (AAGGG)_exp_-associated core haplotype was 11.5 % in patients with medicated parkinsonism and that in population controls is 10.4 %^12^, whereas the allele frequency of the pathogenic expansion in the Finnish population is not known. However, biallelic (AAGGG)_exp_ has shown complete penetrance by eighth decade of age^7^ and a minimum estimate for population frequency can be obtained based on frequencies in the patient cohorts. Such calculations give a frequency of 0.5 %, which is rather similar to that reported for non-Finnish Europeans^1^.

Currently, some 20 genes have been associated with monogenic PD^13^. Our study shows that the biallelic (AAGGG)_exp_ in *RFC1* can be found in patients with PD expanding the phenotypic spectrum of *RFC1* disease. The findings, however, may be specific to the Finnish population and, therefore, other populations need to be examined in order to investigate the potential role of *RFC1* as a monogenic cause of PD.

## Methods

### Patients and controls

Patients with medicated parkinsonism in the provinces of Northern Ostrobothnia, Kainuu and North Karelia were identified in the national medication reimbursement registry of Kela, Finland. DNA was obtained from 569 patients. The controls consisted of 269 geographically matched healthy subjects^12^.

### Molecular genetics

Four polymorphisms defining the (AAGGG)_exp_-associated core haplotype (4-39364970-T-C (rs6844176), 4-39363236-T-C (rs17584703), 4-39327482-G-A (rs11096992) and 4-39317086-A-G (rs2066790) (GRCh38)) were investigated using restriction fragment length polymorphism with FastDigest® *RseI*, *TaaI*, *BseJI* and *Eco105I* (Thermo Fisher Scientific, Waltham, MA, U.S.A.). Core haplotype frequencies were estimated using Arlequin 3.5.2.2 software^14^. A more detailed haplotype of the three patients with PD was constructed using exome sequencing data. PCRs for large (XL-PCR) and complex amplicons were carried out using Phusion High-fidelity DNA polymerase^1^ with HF buffer or Phire Hot-start DNA polymerase (Thermo Fisher Scientific). Flanking multiplex PCR for *FBN1* as a control and *RFC1* was done using TaKaRa Ex Taq Hot Start® polymerase (Takara Bio, Kusatsu, Japan). Fluorescent-labeled repeat-primed PCR (RP-PCR) was carried out for (AAGGG)_exp_, (AAAAG)_exp_, (AAAGG)_exp_ and (ACAGG)_exp_, and the products were analyzed with a GeneScan™ 600LIZ standard (Thermo Fisher Scientific) using capillary sequencer (for detailed reaction conditions, see Supplementary Table 3).

Exome sequencing was carried out as previously^15^ in the patients with biallelic *RFC1* (AAGGG)_exp_ to exclude contributing mutations in known PD genes^13^. Sequencing data were processed using GATK 4.0.6.0 with current Best Practices^TM^ (Broad Institute)^16,17^. Copy number variants were analyzed from the exome data by using XHMM^18^ with lenient parameters^19^.

The number of repeats was determined by using long-range sequencing. Unsheared, purified genomic DNA (3 µg) was used to construct sequencing libraries using the Oxford Nanopore Ligation Sequencing Kit (SQK-LSK109) (Oxford Nanopore Technologies, Oxford, UK) following the manufacturer’s instructions. The enzyme incubation times were doubled with the final AMPure purification incubation of 10 min at 37 °C. The library was loaded onto a flow cell (FLO-MIN106D) on a GridION (Oxford Nanopore Technologies). Target regions were enriched using the adaptive sampling option^20^ on a GridION of high accuracy mode with a bed file assigning the *RFC1* locus along with 58 other loci associated with repeat expansion diseases, and their surrounding regions. Sequencing was performed for 3 days with two additional library loadings. Sequences were basecalled using guppy 4.3.4 during the run on the GridION and aligned to GRCh38 using minimap2.14 (https://github.com/lh3/minimap2) and LAST v1132 (https://gitlab.com/mcfrith/last). Tandem-genotypes v1.3.0 was used to find changes in the length of tandem repeats. When biallelic repeat expansions at the *RFC1* locus were detected, the names of all reads encompassing the *RFC1* locus were picked up using the tandem-genotypes –v option, and FASTA of such reads were generated from FASTQ using seqkit (https://github.com/shenwei356/seqkit). The constructed consensus sequences for both alleles were generated from FASTA files by lamassemble (https://gitlab.com/mcfrith/lamassemble). Detailed repeat analyses were performed using RepeatAnalysisTools (https://github.com/PacificBiosciences/apps-scripts/tree/master/RepeatAnalysisTools).

### Ethics approval and consent to participate

The study protocol was approved by the Ethics Committee of Oulu University Hospital (EETTMK 51/2017) and by Kela (87/522/2017), and written informed consents were given by the patients or their legal caregivers. Written informed consent for the publication of identifiable material was given by patient 1 and 2 (supplementary videos 1 and 2).

### Reporting summary

Further information on research design is available in the Nature Research Reporting Summary linked to this article.

## Supplementary information

Supplementary material

Reporting Summary

Patient 1 before and after levodopa

Patient 2 before levodopa

## Data Availability

Sequence data cannot be made publicly available because of restrictions imposed by the EU and Finnish General Data Protection Regulation (GDPR). Access to sequence data can be applied from the Innovation Agent of the University of Oulu (maarit.jokela@oulu.fi; innovationcentre@oulu.fi). Qualified researchers will be required to complete “Material and data transfer agreement for the transfer of human materials (personal data)”. Genetic variation data have been submitted to ClinVar (SCV002032059). Other data are available within the article or supplementary materials.
