# Supplementary material for: Biallelic expansion in RFC1 as a rare cause of Parkinson’s disease
Source: NPJ Parkinsons Dis. 2022 Jan 10;8:6. doi: 10.1038/s41531-021-00275-7 (PMC8748909; doi:10.1038/s41531-021-00275-7)
Supplement: Supplementary file 1 — Supplementary material [file 41531_2021_275_MOESM1_ESM.pdf]

Biallelic expansion in *RFC1* as a rare cause of Parkinson's disease

Laura Kytövuori*[1,2], Jussi Sipilä[3,4], Hiroshi Doi[5], Anri Hurme-Niiranen[1,2], Ari Siitonen[1,2], Eriko Koshimizu[6], Satoko Miyatake[6,7], Naomichi Matsumoto[6], Fumiaki Tanaka[5], and Kari Majamaa[1,2]

[1]Research Unit of Clinical Neuroscience, Medical Research Center Oulu, Oulu University Hospital and University of Oulu, Oulu, Finland [2]Department of Neurology, Oulu University Hospital, Oulu, Finland, [3]Clinical Neurosciences, University of Turku, Turku, Finland, [4]Department of Neurology, Siun Sote North Karelia Central Hospital, Joensuu, Finland, [5]Department of Neurology and Stroke Medicine, Yokohama City University Graduate School of Medicine, Yokohama, Japan, [6]Department of Human Genetics, Yokohama City University Graduate School of Medicine, Yokohama, Japan, [7]Clinical Genetics Department, Yokohama City University Hospital, Yokohama, Japan

*Corresponding author: Dr. Laura Kytövuori (laura.kytovuori@oulu.fi)

Supplementary material

Supplementary figures 1 and 2

Supplementary tables 1, 2 and 3

Un-cropped, full gel images of Figure 1a and 1b

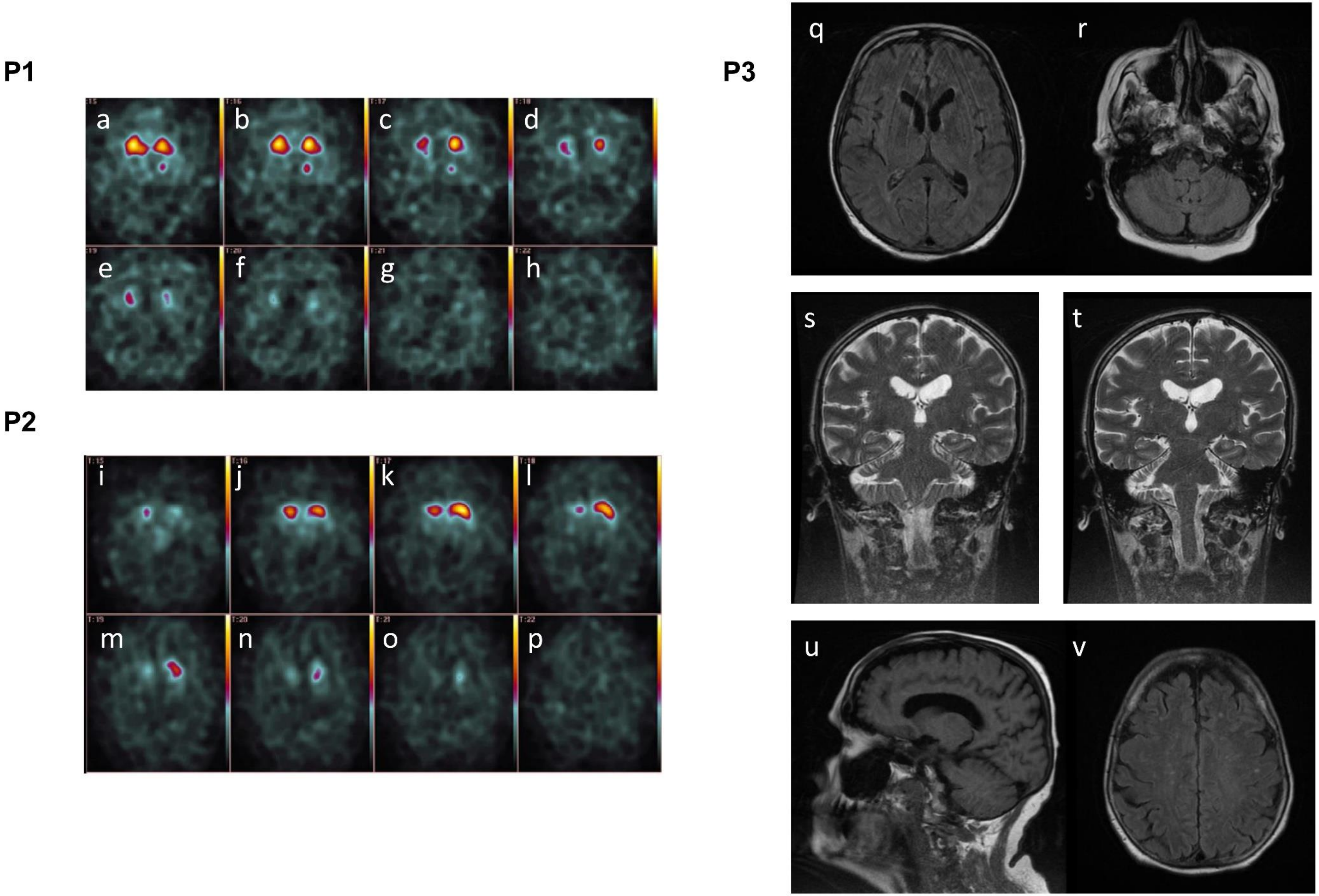

Supplementary figure 1. Beta-CIT-Spect imaging of patient P1 (a-h) and P2 (i-p) and brain magnetic resonance imaging of patient P3 (q-v).

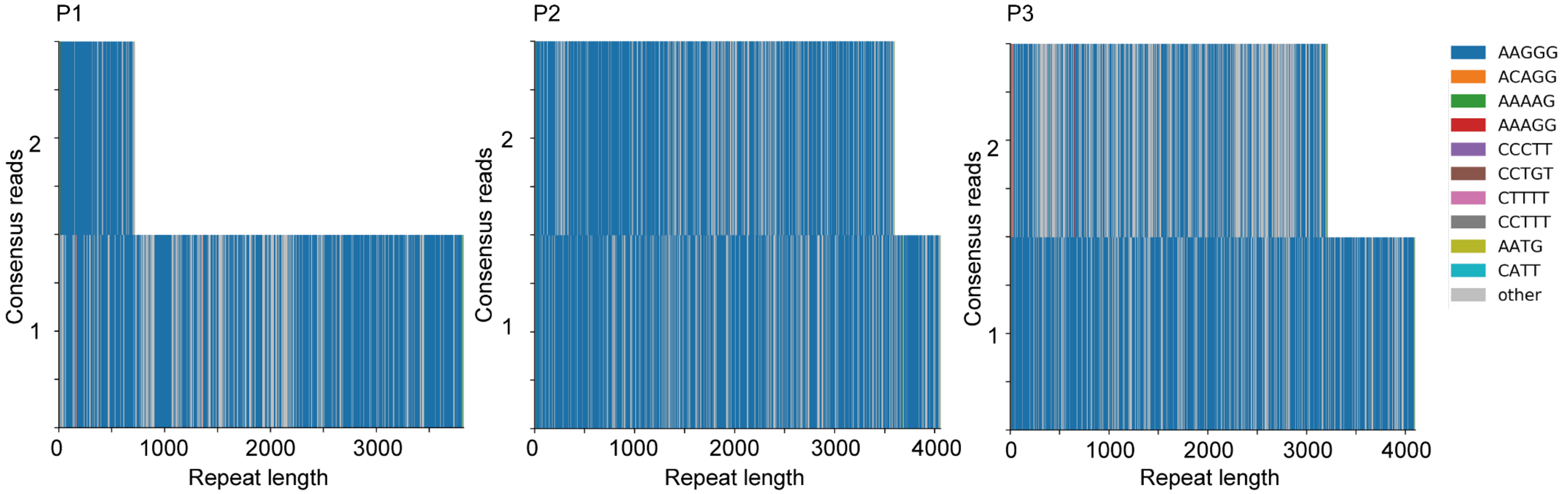

Supplementary figure 2. Consensus reads of the two alleles harboring $(AAGGG)_{exp}$ in patients P1-P3.

Supplementary table 1. Demographic and clinical characteristics of 569 patients with medicated parkinsonism.[1]

	Men (N=344)	Range	Women (N=225)	Range
Age (years)	71.6	39.9-93.4	72.9	42.7-92.2
Age at diagnosis (years)	63.8	32.5-89.4	65.1	36.2-89.2
Duration of symptoms (years)	9	1-36	9	2-24
Tremor at onset (%)	69.5		63.6	
Rigidity at onset (%)	43.6		40.0	
Bradykinesia at onset (%)	47.1		33.3	
Gait instability at onset (%)	28.5		36.3	
Levodopa (%)	84.3		80.0	
First-degree relative with PD (%)	16.3		18.7	
Early-onset PD[2] (%)	21.5		15.3	

[1]Permanent residents in Finland are entitled to full reimbursement for prescription medication in Parkinson's disease or related disorders. In order to obtain the reimbursement a written statement by a neurologist is required confirming the diagnosis. The eligible patients are entered into a reimbursement register that is maintained by the Social Insurance Institution of Finland. Subjects abstracted from the registry are designated here as medicated parkinsonism, because the diagnoses of PD have been made for clinical and not for scientific purposes. Clinical characteristics are patient-reported information. Residents of Northern Ostrobothnia, 329; residents of Kainuu, 79; residents of North Karelia, 161. The values shown are medians. [2]Age at onset < 55 years of age.

Supplementary table 2. *RFC1* repeat length and the number of repeated units $(AAGGG)_{exp}$ determined by Nanopore sequencer. Consensus sequences were generated for both alleles and the number of repeated units was calculated as fragment length (bp)/5.

	Allele	Repeat length (bp)	Repeated units (N)
Patient 1	Allele 1	720	144
	Allele 2	3825	765
Patient 2	Allele 1	3601	720
	Allele 2	4060	812
Patient 3	Allele 1	3215	643
	Allele 2	4099	820

Supplementary table 3. PCR-primers and reaction conditions.

***RFC1* primers and protocols**

**Primers for *RFC1* haplotyping**[12]	**5′ → 3′**	**Final concentration**
RFC1_HT1_F (rs2066790)	ACCACCACGCCATCACAACCC	0.5 µM
RFC1_HT1_R (rs2066790)	TCGGGCGATCAGGTTTACTTGGG	0.5 µM
RFC1_HT2_F (rs11096992)	ACACACACTATGGGGCAGGCT	0.5 µM
RFC1_HT2_R (rs11096992)	AGGCGGCCTCTAAATCAAAAGAGA	0.5 µM
RFC1_HT3_F (rs17584703)	AGGGGCCTTAGAGGCCAAGTTC	0.5 µM
RFC1_HT3_R (rs17584703)	AGACAGCTCTTGCCCTGGGAGC	0.5 µM
RFC1_HT4_F (rs6844176)	GCCTAACATGCCAGGCACCAGA	0.5 µM
RFC1_HT4_R (rs6844176)	CTGGGAAACGTTGCAGGCCTTTG	0.5 µM

**Primers for *RFC1* XL-PCR**[1]	**5′ → 3′**	**Final concentration**
RFC1_XLF	TCAAGTGATACTCCAGCTACACCGTTGC	0.5 µM
RFC1_XLR	GTGGGAGACAGGCCAATCACTTCAG	0.5 µM

**Primers for multiplex PCR**[1,12]	**5′ → 3′**	**Final concentration**
RFC1_F	TCAAGTGATACTCCAGCTACACCGTTGC	0.5 µM
RFC1_R	GTGGGAGACAGGCCAATCACTTCAG	0.5 µM
FBN1_F	GGCCATCTCTTCCTCTTCTTCTT	0.25 µM
FBN1_R	TGATTTCCGCCAGGTAAGGT	0.25 µM

Supplementary table 3 continues.

**Primers for *RFC1* RP-PCR**[1]	**5′ → 3′**	**Final concentration**
RFC1_Fw_FAM	TCAAGTGATACTCCAGCTACACCGT	0.5 µM
RFC1_anchor (=M13linker)	CAGGAAACAGCTATGACC	0.5 µM
RFC1_Rv1_AAAAG11	CAGGAAACAGCTATGACCAACAGAGCAAGACTCTGTTTCAAAAAAGAAAAGAAAAGAAAAGAAAA	0.05 µM
RFC1_Rv2_AAAAG11	CAGGAAACAGCTATGACCAACAGAGCAAGACTCTGTTTCAAAAAGAAAAGAAAAGAAAAGAAAA	0.05 µM
RFC1_Rv3_AAAAG11	CAGGAAACAGCTATGACCAACAGAGCAAGACTCTGTTTCAAAAGAAAAGAAAAGAAAAGAAAA	0.05 µM
RFC1_Rv1_AAAGG_exp	CAGGAAACAGCTATGACCAACAGAGCAAGACTCTGTTTCAAAAAAGGAAAGGAAAGGAAAGGAAA	0.05 µM
RFC1_Rv2_AAAGG_exp	CAGGAAACAGCTATGACCAACAGAGCAAGACTCTGTTTCAAAAAGGAAAGGAAAGGAAAGGAAA	0.05 µM
RFC1_Rv3_AAAGG_exp	CAGGAAACAGCTATGACCAACAGAGCAAGACTCTGTTTCAAAAGGAAAGGAAAGGAAAGGAAA	0.05 µM
RFC1_Rv1_AAGGG_exp	CAGGAAACAGCTATGACCAACAGAGCAAGACTCTGTTTCAAAAAAGGGAAGGGAAGGGAAGGGAA	0.05 µM
RFC1_Rv2_AAGGG_exp	CAGGAAACAGCTATGACCAACAGAGCAAGACTCTGTTTCAAAAAGGGAAGGGAAGGGAAGGGAA	0.05 µM
RFC1_Rv3_AAGGG_exp	CAGGAAACAGCTATGACCAACAGAGCAAGACTCTGTTTCAAAAGGGAAGGGAAGGGAAGGGAA	0.05 µM
RFC1_Rv1_ACAGG_exp	CAGGAAACAGCTATGACCAACAGAGCAAGACTCTGTTTCAAAAACAGGACAGGACAGGACAGGAC	0.05 µM
RFC1_Rv2_ACAGG_exp	CAGGAAACAGCTATGACCAACAGAGCAAGACTCTGTTTCAAAACAGGACAGGACAGGACAGGAC	0.05 µM
RFC1_Rv3_ACAGG_exp	CAGGAAACAGCTATGACCAACAGAGCAAGACTCTGTTTCAAACAGGACAGGACAGGACAGGAC	0.05 µM

Supplementary table 3 continues.

Reaction conditions

**Haplotyping**[12]	**PCR additives**	**DNA Polymerase**	**Denaturation 1**	**Denaturation 2**	**Annealing**	**Extension**	**Cycles**	**Final extension**
HT1	3 % DMSO	Phire Hot Start II	98 °C, 45 s	98 °C, 5 s	66 °C, 5 s	72 °C, 7 s	30	72 °C, 60 s
HT2	3 % DMSO	Phire Hot Start II	98 °C, 45 s	98 °C, 5 s	66 °C, 5 s	72 °C, 7 s	30	72 °C, 60 s
HT3	3 % DMSO	Phire Hot Start II	98 °C, 45 s	98 °C, 5 s	66 °C, 5 s	72 °C, 7 s	30	72 °C, 60 s
HT4	3 % DMSO	Phire Hot Start II	98 °C, 45 s	98 °C, 5 s	67 °C, 5 s	72 °C, 6 s	30	72 °C, 60 s

**XL-PCRs**	**PCR additives**	**DNA Polymerase**	**Denaturation 1**	**Denaturation 2**	**Annealing**	**Extension**	**Cycles**	**Final extension**
XL-Phire[12]	3 % DMSO	Phire Hot Start II	98 °C, 45 s	98 °C, 5 s	67 °C, 5 s	72 °C, 5 s/70 s	33	72 °C, 60 s/75 s
XL-Phusion[1]	3 % DMSO	Phusion HF	98 °C, 3 min	98 °C, 10 s	65 °C, 15 s decrease 0.5 °C/cycle	72 °C, 3 min	18	↓
				→	57 °C, 15 s	72 °C, 3 min	18	72 °C, 5 min

FBN1* & *RFC1[12]	**DNA Polymerase**	**Denaturation 1**	**Denaturation 2**	**Annealing**	**Extension**	**Cycles**	**Final extension**
Multiplex	Takara Ex Taq	95 °C, 4 min	95 °C, 30 s	59 °C, 30 s	72 °C, 60 s	35	72 °C, 5 min

**RP-PCR**[12]	**PCR additives**	**DNA Polymerase**	**Denaturation 1**	**Denaturation 2**	**Extension**	**Cycles**	**Final extension**
RP-PCR	3 % DMSO	Phusion Flash	98 °C, 3 min	98 °C, 10 s	72 °C, 60 s	35	72 °C, 5 min

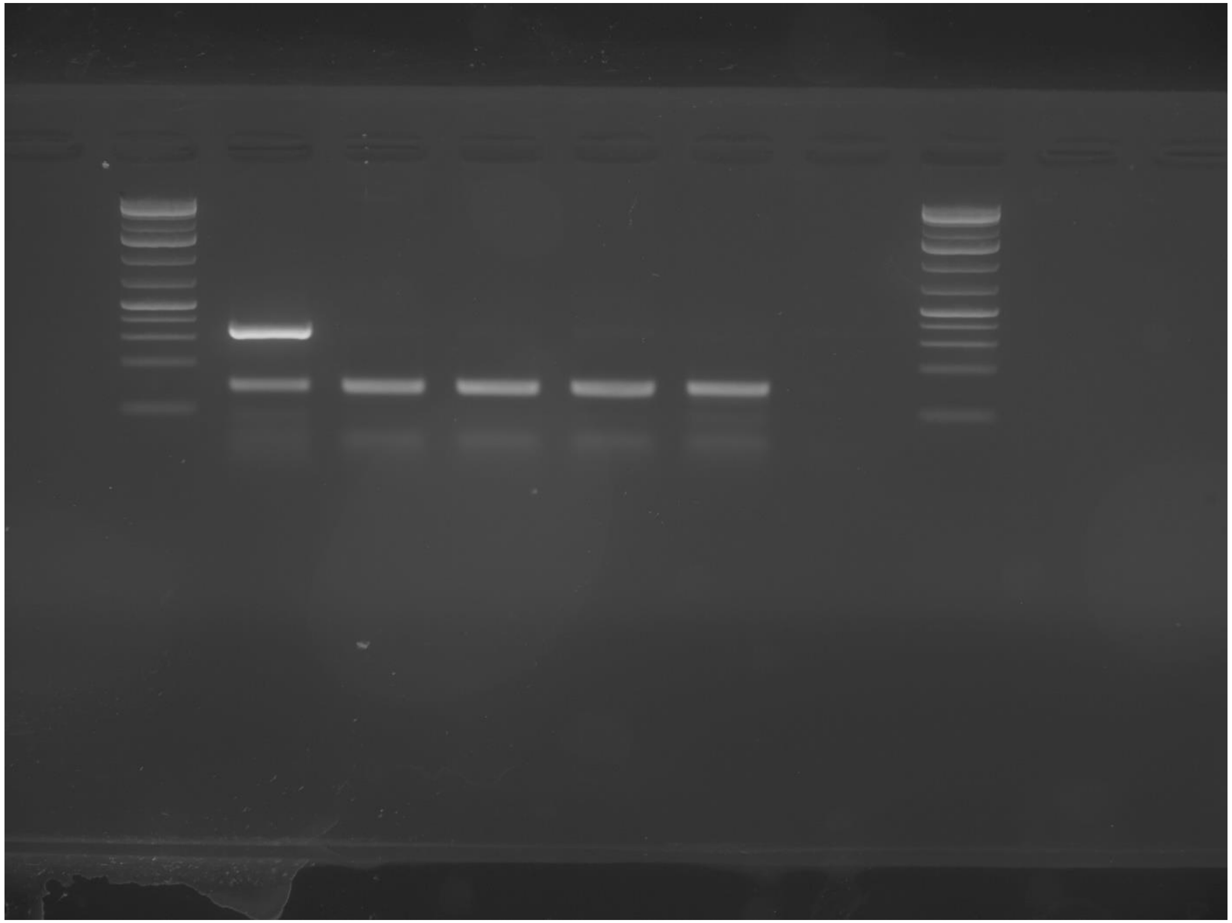

Additional figure. Un-cropped, full version of Figure 1a. Multiplex PCR of *RFC1* and *FBN1* shows no *RFC1* PCR product in the region of interest in the three patients with PD or CANVAS. Lane 1, control; lanes 2-4, P1-P3; lane 5, patient with CANVAS and biallelic $(AAGGG)_{exp}$; lane 6, H_2O. The gel derives from the same experiment.

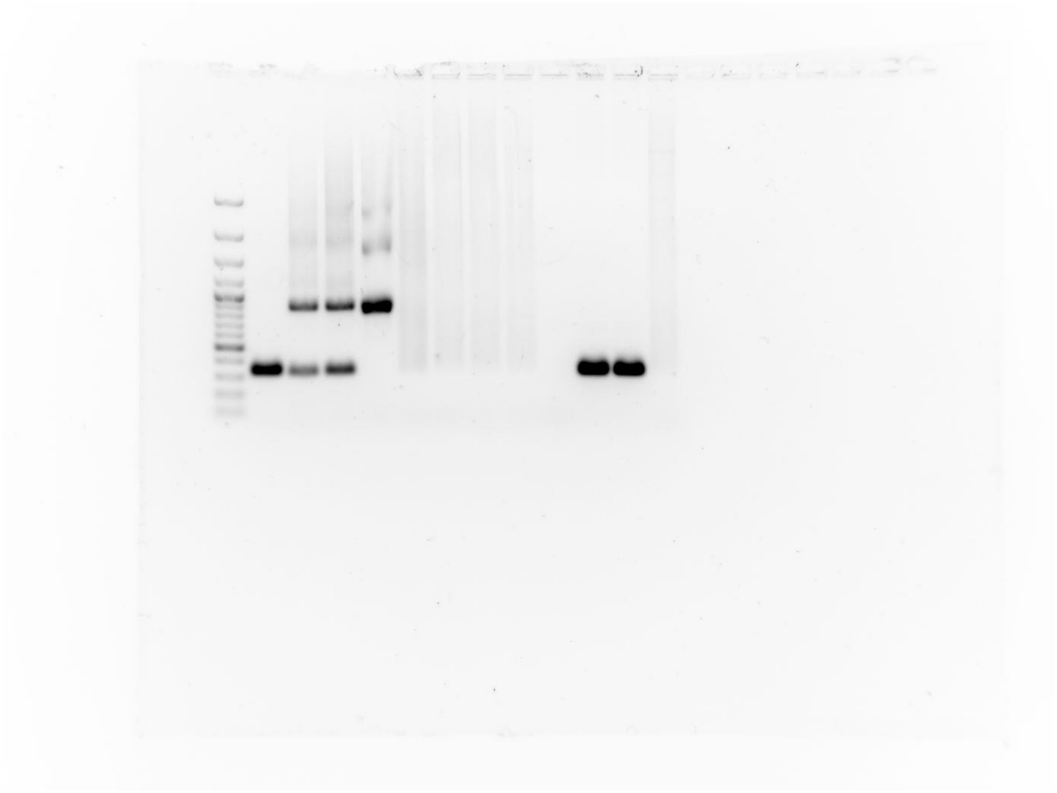

Additional figure. Un-cropped, full version of Figure 1b. Marker, GeneRuler 100 bp Plus DNA Ladder (Thermo Fisher Scientific); Lanes 1-4, healthy controls with normal fragment size variation; lanes 5-7, patients 1-3; lane 8, patient with CANVAS and biallelic $(AAGGG)_{exp}$; lane 9, H_2O; lanes 10-11, controls; lane 12, patient with CANVAS and biallelic $(AAGGG)_{exp}$; lane 13, H_2O. The gel derives from the same experiment.
