# Supplementary material for: Biallelic expansion in RFC1 as a rare cause of Parkinson’s disease
Source: NPJ Parkinsons Dis. 2022 Jan 10;8:6. doi: 10.1038/s41531-021-00275-7 (PMC8748909; doi:10.1038/s41531-021-00275-7)
Supplement: Supplementary file 2 — Reporting Summary [file 41531_2021_275_MOESM2_ESM.pdf]

nature portfolio

Corresponding author(s): Laura Kytövuori

Last updated by author(s): Dec 17, 2021

Reporting Summary

Nature Portfolio wishes to improve the reproducibility of the work that we publish. This form provides structure for consistency and transparency in reporting. For further information on Nature Portfolio policies, see our Editorial Policies and the Editorial Policy Checklist.

Statistics

For all statistical analyses, confirm that the following items are present in the figure legend, table legend, main text, or Methods section.

n/a	Confirmed	
☐	☒	The exact sample size (*n*) for each experimental group/condition, given as a discrete number and unit of measurement
☒	☐	A statement on whether measurements were taken from distinct samples or whether the same sample was measured repeatedly
☒	☐	The statistical test(s) used AND whether they are one- or two-sided<br>*Only common tests should be described solely by name; describe more complex techniques in the Methods section.*
☒	☐	A description of all covariates tested
☒	☐	A description of any assumptions or corrections, such as tests of normality and adjustment for multiple comparisons
☒	☐	A full description of the statistical parameters including central tendency (e.g. means) or other basic estimates (e.g. regression coefficient) AND variation (e.g. standard deviation) or associated estimates of uncertainty (e.g. confidence intervals)
☒	☐	For null hypothesis testing, the test statistic (e.g. *F*, *t*, *r*) with confidence intervals, effect sizes, degrees of freedom and *P* value noted<br>*Give P values as exact values whenever suitable.*
☒	☐	For Bayesian analysis, information on the choice of priors and Markov chain Monte Carlo settings
☒	☐	For hierarchical and complex designs, identification of the appropriate level for tests and full reporting of outcomes
☒	☐	Estimates of effect sizes (e.g. Cohen's *d*, Pearson's *r*), indicating how they were calculated

Our web collection on statistics for biologists contains articles on many of the points above.

Software and code

Policy information about availability of computer code

Data collection	No software was used.
Data analysis	No custom algorithms or software were used.

For manuscripts utilizing custom algorithms or software that are central to the research but not yet described in published literature, software must be made available to editors and reviewers. We strongly encourage code deposition in a community repository (e.g. GitHub). See the Nature Portfolio guidelines for submitting code & software for further information.

Data

Policy information about availability of data

All manuscripts must include a data availability statement. This statement should provide the following information, where applicable:

- Accession codes, unique identifiers, or web links for publicly available datasets
- A description of any restrictions on data availability
- For clinical datasets or third party data, please ensure that the statement adheres to our policy

Sequence data cannot be made publicly available because of restrictions imposed by the EU and Finnish General Data Protection Regulation (GDPR). Access to sequence data can be applied from the Innovation Agent of the University of Oulu (maarit.jokela@oulu.fi; innovationcentre@oulu.fi). Qualified researchers will be required to complete “Material and data transfer agreement for the transfer of human materials (personal data)". Genetic variation data have been submitted to ClinVar (SCV002032059). Other data are available within the article or supplementary materials.

Field-specific reporting

Please select the one below that is the best fit for your research. If you are not sure, read the appropriate sections before making your selection.

☒ Life sciences ☐ Behavioural & social sciences ☐ Ecological, evolutionary & environmental sciences

For a reference copy of the document with all sections, see nature.com/documents/nr-reporting-summary-flat.pdf

Life sciences study design

All studies must disclose on these points even when the disclosure is negative.

Sample size	569 patients (344 men) with medicated parkinsonism participated in our study. 269 healthy controls.
Data exclusions	No patients were excluded.
Replication	Not relevant.
Randomization	No experimental groups were created.
Blinding	Not relevant.

Reporting for specific materials, systems and methods

We require information from authors about some types of materials, experimental systems and methods used in many studies. Here, indicate whether each material, system or method listed is relevant to your study. If you are not sure if a list item applies to your research, read the appropriate section before selecting a response.

Materials & experimental systems

n/a	Involved in the study	
☒	☐	Antibodies
☒	☐	Eukaryotic cell lines
☒	☐	Palaeontology and archaeology
☒	☐	Animals and other organisms
☐	☒	Human research participants
☒	☐	Clinical data
☒	☐	Dual use research of concern

Methods

n/a	Involved in the study	
☒	☐	ChIP-seq
☒	☐	Flow cytometry
☒	☐	MRI-based neuroimaging

Human research participants

Policy information about studies involving human research participants

Population characteristics	569 patients (344 men) with medicated parkinsonism participated in our study. Median age was 71.6 yrs in men (range, 39-93) and 72.9 yrs in women (range, 42-92). Age of onset was 63.8 yrs in men, 65.1 yrs in women.
Recruitment	Patients were ascertained from the provinces of Northern Ostrobothnia, Kainuu and North Karelia with a total population of 647,462 (Statistics Finland; https://www.stat.fi/index_en.html). All patients with Parkinson´s disease are entitles to reimbursement of medicine expenses by the Social Insurance Institute of Finland (Kela). The diagnosis of PD has been made by a neurologist. There were 2142 patients with an entitlement to reimbursement in the three provinces on September 30 2018. 569 patients volunteered to participate in the study. A blood sample was obtained at the local health care unit.
Ethics oversight	The study protocol was approved by the Ethics Committee of Oulu University Hospital (EETTMK 51/2017) and by Kela (87/522/2017), and written informed consents were given by the patients or their legal caregivers. Written informed consent was obtained from identifiable subjects.

Note that full information on the approval of the study protocol must also be provided in the manuscript.
